# Grey zone amyloid burden affects memory function: the SCIENCe project

**DOI:** 10.1007/s00259-020-05012-5

**Published:** 2020-09-04

**Authors:** J. L. Ebenau, S. C. J. Verfaillie, K. A. van den Bosch, T. Timmers, L. M. P. Wesselman, M. van Leeuwenstijn, H. Tuncel, S. V. S. Golla, M. M. Yaqub, A. D. Windhorst, N. D. Prins, F. Barkhof, P. Scheltens, W. M. van der Flier, B. N. M. van Berckel

**Affiliations:** 1 Alzheimer Centre, Department of Neurology, Vrije Universiteit Amsterdam, Amsterdam UMC, Amsterdam, The Netherlands; 2 Department of Radiology & Nuclear Medicine, Amsterdam Neuroscience, Vrije Universiteit Amsterdam, Amsterdam UMC, Amsterdam, The Netherlands; 3 Brain Research Centre, Amsterdam, The Netherlands; 4 UCL Institutes of Neurology and Healthcare Engineering, London, UK; 5 Department of Epidemiology & Biostatistics, Vrije Universiteit Amsterdam, Amsterdam UMC, Amsterdam, The Netherlands

**Keywords:** [^18^F] florbetapir, Amyloid, Subjective cognitive decline, Grey zone, Cognition

## Abstract

**Purpose:**

To determine thresholds for amyloid beta pathology and evaluate associations with longitudinal memory performance with the aim to identify a grey zone of early amyloid beta accumulation and investigate its clinical relevance.

**Methods:**

We included 162 cognitively normal participants with subjective cognitive decline from the SCIENCe cohort (64 ± 8 years, 38% F, MMSE 29 ± 1). Each underwent a dynamic [^18^F] florbetapir PET scan, a T1-weighted MRI scan and longitudinal memory assessments (RAVLT delayed recall, *n* = 655 examinations). PET scans were visually assessed as amyloid positive/negative. Additionally, we calculated the mean binding potential (BP_ND_) and standardized uptake value ratio (SUVr_50–70_) for an a priori defined composite region of interest. We determined six amyloid positivity thresholds using various data-driven methods (resulting thresholds: BP_ND_ 0.19/0.23/0.29; SUVr 1.28/1.34/1.43). We used Cohen’s kappa to analyse concordance between thresholds and visual assessment. Next, we used quantiles to divide the sample into two to five subgroups of equal numbers (median, tertiles, quartiles, quintiles), and operationalized a grey zone as the range between the thresholds (0.19–0.29 BP_ND_/1.28–1.43 SUVr). We used linear mixed models to determine associations between thresholds and memory slope.

**Results:**

As determined by visual assessment, 24% of 162 individuals were amyloid positive. Concordance with visual assessment was comparable but slightly higher for BP_ND_ thresholds (range kappa 0.65–0.70 versus 0.60–0.63). All thresholds predicted memory decline (range beta − 0.29 to − 0.21, all *p* < 0.05). Analyses in subgroups showed memory slopes gradually became steeper with higher amyloid load (all *p* for trend < 0.05). Participants with a low amyloid burden benefited from a practice effect (i.e. increase in memory), whilst high amyloid burden was associated with memory decline. Memory slopes of individuals in the grey zone were intermediate.

**Conclusion:**

We provide evidence that not only high but also grey zone amyloid burden subtly impacts memory function. Therefore, in case a binary classification is required, we suggest using a relatively low threshold which includes grey zone amyloid pathology.

## Introduction

The presence of pathological amyloid beta depositions is one of the hallmarks of Alzheimer’s disease (AD) and amyloid pathology is thought to play an important role in its pathophysiology [1, 2]. Indeed, high amyloid burden in cognitively normal individuals is associated with a greater risk of cognitive decline, particularly of memory function [3–9]. Furthermore, individuals with subjective cognitive decline (SCD) are more often amyloid positive than the general population and are at increased risk of cognitive decline and dementia [10, 11]. Therefore, individuals with SCD form an ideal population to study the effects of ‘early’ amyloid deposition on cognition.

Amyloid beta pathology can be assessed in vivo by [^18^F] florbetapir positron emission tomography (PET) using visual assessment in a dichotomous manner, i.e. positive versus negative [12]. However, the accuracy depends on the expertise of the trained reader [13], and visual assessment of scans with early amyloid accumulation can be challenging. Classification of amyloid positivity can also be determined using a threshold as an alternative to visual assessment. The standardized uptake value ratio (SUVr) is a widely used method for estimating amyloid burden semi-quantitatively using a static scan procedure. Dynamic scanning allows for calculation of binding potential (BP_ND_) which provides a more exact quantification of specific binding to amyloid beta [14]. BP_ND_ has been shown to be less prone to overestimation compared with SUVr and is more reliable when studying early amyloid accumulation [15–17]. So far, different SUVr thresholds for [^18^F] florbetapir have been proposed [18–23], but these thresholds are highly variable (range SUVr thresholds 1.08–1.34). BP_ND_ thresholds have not been published yet.

Dichotomizing amyloid burden into a negative and positive status can be useful in clinical and research settings, but it disregards the potential significance of early (pathological) amyloid accumulation [24]. Recent studies show that even in individuals that are initially labelled as amyloid negative, the amyloid accumulation slope is associated with memory decline [25, 26]. It is uncertain whether this suggests that current thresholds are simply too high and lower thresholds would be able to correctly classify individuals, or that there is a more gradual association between memory decline and amyloid burden. The latter would point towards a ‘dose-dependent risk’ with a grey zone amyloid burden reflecting an at-risk state for AD.

In the current study, we aimed to define thresholds for amyloid positivity using data-driven methods based on both SUVr and BP_ND_. Subsequently, we compared each of these classifications with visual assessment of amyloid positivity and determined associations with memory function over time. In addition, we identified a ‘grey zone’ of amyloid burden in cognitively normal individuals, and investigated its clinical significance, by exploring the nature of the relationship between amyloid levels in the subthreshold range and memory slope.

## Method

### Population

We included 162 cognitively normal participants with SCD from the Subjective Cognitive Impairment Cohort (SCIENCe) within the Amsterdam Dementia Cohort at the Alzheimer Centre Amsterdam [27, 28]. All subjects with [^18^F] florbetapir PET, magnetic resonance imaging (MRI) scan and cognitive data available were included. One hundred and fifty-two participants were referred to the memory clinic by their general physician, a neurologist or a geriatrician, and underwent an extensive standardized diagnostic workup that included a neurologic and neuropsychological examination, laboratory testing and brain MRI [28, 29]. In a consensus meeting, participants were labelled SCD when cognitive performance appeared within normal limits compared with peers, and criteria were not met for mild cognitive impairment (MCI), dementia or other neurological or psychiatric diseases that could possibly cause cognitive complaints. At annual follow-up visits, neuropsychological testing was repeated and diagnoses were re-evaluated. In addition, 10 participants were included as research participants via the Dutch Brain Research Registry (hersenonderzoek.nl). They also experienced cognitive complaints in absence of a diagnosis of MCI or dementia, and received the same baseline workup.

### Neuropsychological assessment

We previously showed that the relationship between amyloid burden and cognitive decline was strongest for the memory domain, especially for the Rey auditory verbal learning task (RAVLT) delayed recall [7]. Therefore, for this study, we used the RAVLT delayed recall as a measure for memory function. We used visits conducted before as well as after the PET scan to accurately estimate the memory slope, resulting in longitudinal cognitive data covering 3.8 ± 3.1 years. Concurrent time points were defined as the visit closest to the PET scan date (median − 0.19 (IQR − 0.38–0.14 years)). We used two different versions of the RAVLT, between which we alternated at the annual follow-up visits. In total, 655 neuropsychological examinations of 162 participants were available (149 ≥ 2 visits; range 1–10; median 3 visits).

### Questionnaires

Within the SCIENCe cohort, a number of questionnaires are administered to evaluate subjective cognitive complaints, mental health, instrumental activities of daily living and lifestyle [27]. For this study, we used the cognitive change index (CCI, 20 questions, range 0–80) to quantify the degree of subjective cognitive complaints. We additionally used the geriatric depression scale (GDS, 15 questions, range 0–15) to evaluate depressive symptoms. For both questionnaires, a higher score reflects more severe symptoms.

### PET acquisition and image analysis

PET scans were acquired on an Ingenuity PET-CT (*n* = 115) or a Gemini TF PET-CT (*n* = 47; Philips, Best, the Netherlands) scanner. Dynamic PET emission scans of 90 min (*n* = 137) were obtained starting directly after tracer injection of approximately 370 M Becquerel (MBq) [^18^F] florbetapir. During the course of the study, our group showed that the scan duration could be reduced without compromising the reliability of results [14]. Therefore, the more recent scans (*n* = 21) had a duration of 70 min. Furthermore, in four participants, the scan was terminated early (three after 60 min, one after 79 min) due to participant-related issues. These scans were still used since they had an uninterrupted 60 min of scanning [14]. Head movement when lying in the camera was monitored with laser beams, and if necessary, the position of the head was corrected. Data were reconstructed with a standard LOR RAMLA reconstruction algorithm into 22 frames, and images were corrected for scatter, random coincidences, attenuation, decay and dead time. Images were reconstructed with a matrix size of 128 × 128 × 90 and a voxel size of 2 × 2 × 2 mm^3^. Isotropic 3-dimensional T1-weighted MR images (GE Discovery MR750 3 T (*n* = 58), PETMR 3 T (*n* = 71), Signa 1.5 T (*n* = 6), Signa 3 T (*n* = 2), Titan 3 T (*n* = 24) and external scan (*n* = 1)) were co-registered to PET images using the Vinci software (Max Planck Institute, Cologne, Germany). Regions of interest (ROIs) were defined on the co-registered MRI using the Hammers probability atlas [30] in PVElab. Receptor parametric mapping (RPM) was used to generate BP_ND_ images with cerebellar grey matter as a reference region [16, 31, 14, 17]. We extracted BP_ND_ and SUVr_50–70_ values in the following a priori defined regions: orbitofrontal, temporal, parietal, anterior cingulate, posterior cingulate and precuneus [21]. An SUVr time interval of 50–70 min post-injection was chosen because this is commonly used, and our group showed before that SUVr becomes constant from 40 min onward [14, 18]. We subsequently averaged the values of the a priori defined regions into one volume weighted mean cortical BP_ND_ or SUVr value. The difference in time between MRI and PET was generally within 1 year (median time difference 0.22 years (IQR − 0.49–0.55)).

### Threshold derivation

SUV images were visually assessed as ‘positive’ or ‘negative’ by a trained and experienced nuclear medicine physician (BvB) who was blinded for clinical information, based on standards provided by the manufacturer [32]. Next, we used different data-driven methods to obtain thresholds for amyloid positivity for both BP_ND_ and SUVr. First we used the R studio function normalmixEM to fit Gaussian mixture models (GMM) with 1–9 components. Bayesian information criterion (BIC) indicated a model with 2 components as being the most optimal fit to our data. A threshold was derived representing the mean of the calculated mu of both components. The calculated thresholds were similar when we used the proportions derived from visual assessment (24% and 76%) as a starting value for mixture weights. This resulted in cut-off points of 0.23 (BP_ND_) and 1.34 (SUVr).

**Fig. 1:**
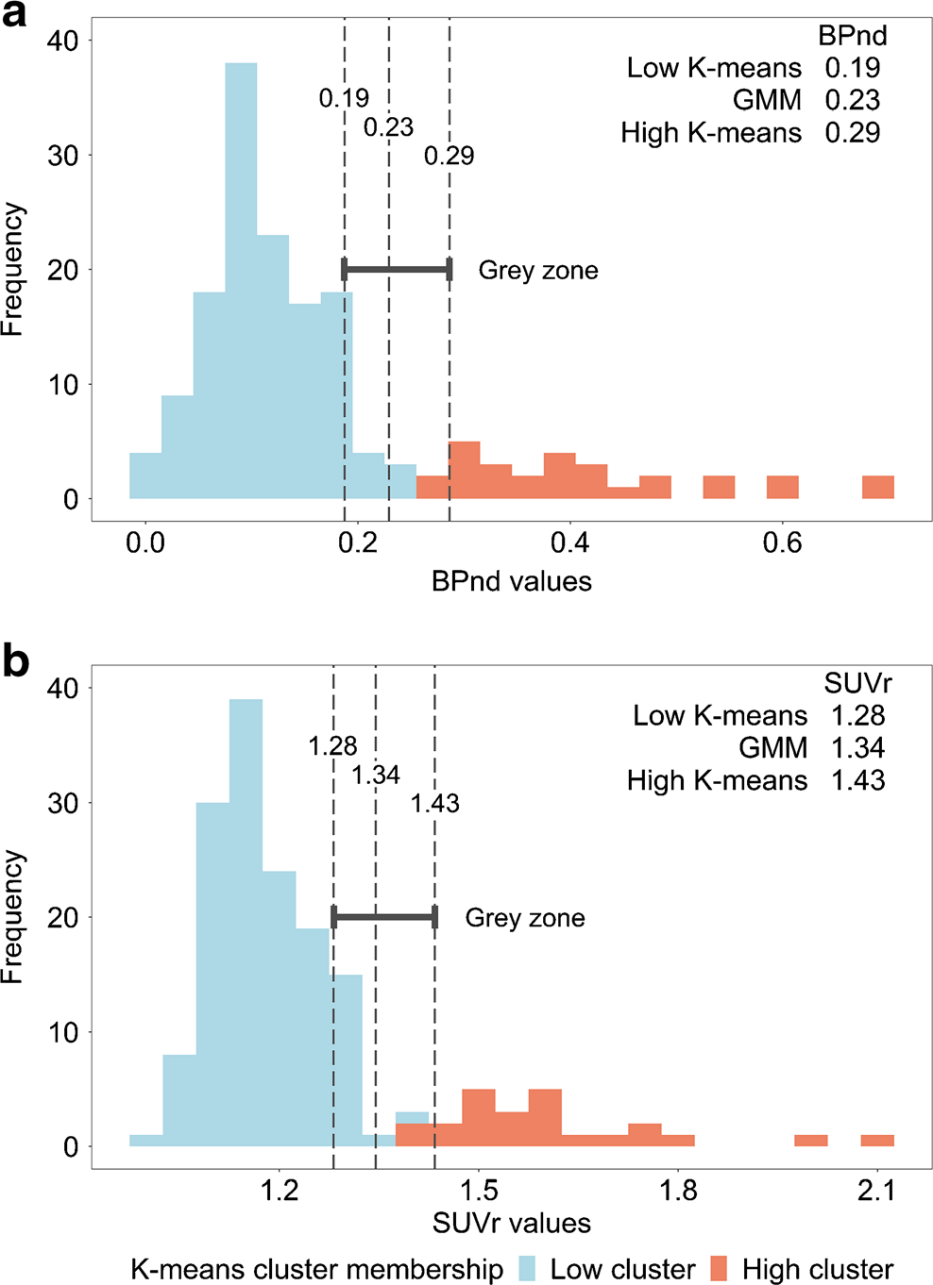
Visualization of thresholds and grey zone. Two frequency histograms containing all mean BP_ND_ values (**a**) and SUVr values (**b**), with K-means cluster membership visualized by different colours (blue and red). The dashed lines represent the 6 different thresholds (for BP_ND_: 0.19, 0.23 and 0.29, for SUVr: 1.28, 1.34 and 1.43). The grey zone was operationalized as the range between the lowest and the highest thresholds derived through K-means clustering. For BP_ND_, 121 participants had an amyloid burden lower than the low K-means threshold, 15 participants had grey zone amyloid burden and 26 participants had an amyloid burden higher than the high K-means threshold. For SUVr, the numbers were 125, 15 and 22 respectively

Next, we used K-means clustering. We assumed the data consisted of two clusters. We derived two cut-off values, the first representing the 90th percentile of the cluster with low amyloid burden, and the second representing the 10th percentile of the cluster with high amyloid burden. The cut-off values were purely data-driven, and information about visual assessment of scans was not used for these thresholds. This resulted in a low threshold (0.19 BP_ND_ and 1.28 SUVr), and a high threshold (0.29 BP_ND_ and 1.43 SUVr). Subsequently, we took the area between the lower and higher thresholds derived by K-means clustering to operationalize a grey zone. Figure 1 shows a summary of all derived thresholds and visualizes the predefined grey zone.

### Statistics

We used *t* test, Mann-Whitney *U* and chi-square where appropriate to compare demographic measures between amyloid positive and amyloid negative groups, based on visual assessment. We used Cohen’s kappa to determine the degree of concordance between visual assessment on the one hand and the six thresholds on the other hand. We used linear mixed models (LMM) to assess the associations between amyloid status (visual and data-driven) and memory slopes. Separate models were run for each of the seven ways of defining amyloid positivity. Amyloid status, time and the interaction between amyloid status and time were included as independent variables, age, sex, education and scanner type were included as covariates, and scores on the RAVLT delayed memory task were used as dependent variables. Intercept and time were included as random factors, as this resulted in a better fit. Using these models, we estimated the annual change over time for both a negative and a positive amyloid status. We compared models based on betas, *p* values and Akaike information criterion (AIC).

Subsequently, using an increasing number of quantiles, we divided the sample into two, three, four and five equal-sized distributions (i.e. subgroups) to explore whether there is a gradual association between amyloid burden and memory slope. We subsequently used LMM to estimate memory slopes for each subgroup. Separate analyses were run for each quantile-based division. Subgroups (entered as dummies), time and the interaction between subgroups and time were included as independent variables, age, sex, education and scanner type were included as covariates, and RAVLT delayed recall score was used as dependent variable. In addition, we ran models including subgroups as continuous variables, and present the resulting *p* value for trend.

All analyses were done using SPSS version 26 and R studio version 1.1.463. For the estimated trends, we used the R studio function of emtrends. *p* values < 0.05 were considered significant.

## Results

### Baseline demographics

At baseline, individuals were on average 64 ± 8 years old, 63 (39%) were female and MMSE was 29 ± 1 (Table 1). Among 162 individuals, 38 (24%) were amyloid positive as defined by visual assessment. Amyloid positive (A+) individuals were on average older and more often APOE4 carrier than amyloid negative (A−) individuals.

**Table 1:** Baseline demographics by amyloid status based on visual assessment

	Total *n* = 162	Amyloid negative *n* = 124	Amyloid positive *n* = 38
Age, mean (SD)	64 (8)	63 (8)	68 (8)*
Sex, *n* female (%)	63 (39%)	47 (38%)	16 (42%)
Education, mean (SD)^a^	6 (1)	6 (1)	6 (1)
APOE4 status, *n* carrier (%)	54 (36%)	31 (26%)	23 (68%)*
CCI, mean (SD)^a,b^	21.8 (14.5)	21.4 (15.0)	23.0 (13.1)
GDS, mean (SD)^a,b^	2.4 (2.0)	2.5 (2.1)	2.3 (1.9)
MMSE, mean (SD)^a,b^	28.9 (1.2)	28.9 (1.2)	28.7 (1.2)
RAVLT delayed, mean (SD)^b^	9.1 (3.0)	9.3 (3.0)	8.3 (3.2)
Amyloid load (BP_ND_), mean (SD)	0.16 (0.13)	0.11 (0.06)	0.33 (0.18)*
Amyloid load (SUVr), mean (SD)	1.24 (0.18)	1.17 (0.08)	1.45 (0.26)*

Amyloid status was determined by visual assessment of the [^18^F] florbetapir PET scan. Education is rated using the Dutch Verhage system [33]. Amyloid load represents the volume-weighted mean cortical value in a composite region of a priori defined regions (orbitofrontal, temporal, parietal, anterior cingulate, posterior cingulate and precuneus), with cerebellar grey matter as reference region. CCI and GDS test scores were available for 159 and 96 participants respectively

*CCI* cognitive change index, *SCF* subjective cognitive functioning, *GDS* geriatric depression scale, *MMSE* mini-mental state examination, *RAVLT* Rey auditory verbal learning task, *BP*_*ND*_ binding potential, *SUVr* standardized uptake value ratio

^a^Mann-Whitney *U* test. All other analyses were performed using *t* test and Chi-square

^b^Score on concurrent test

^*^p < 0.01 for difference between amyloid negative and positive individuals

We presented thresholds, frequency and kappa values in Table 2. When we applied the different thresholds, the amyloid positivity rates ranged from 26 (16%) to 41 (25%) for BP_ND_ thresholds, and from 22 (14%) to 37 (23%) for SUVr thresholds. For BP_ND_ as well as SUVr, the low K-means threshold resulted in the highest percentage of A+ individuals, and the high K-means threshold in the lowest percentage of A+ individuals. The grey zone, operationalized as the range between the lowest and highest K-means thresholds, consisted of 9% of individuals for both BP_ND_ and SUVr. Cohen’s kappa showed that there was substantial concordance between visual assessment and each of the six thresholds. Upon visual inspection, concordance was highest for BP_ND_ thresholds, but confidence intervals overlapped.

**Table 2:** Cut-off values for different methods

	Derivation method	Threshold	*n* positive (%)	Kappa (95% CI)
Visual assessment			38 (24%)	
BP_ND_	Low K-means	0.19	41 (25%)	0.65 (0.51–0.79)
	GMM	0.23	30 (19%)	0.70 (0.57–0.84)
	High K-means	0.29	26 (16%)	0.65 (0.51–0.80)
SUVr	Low K-means	1.28	37 (23%)	0.60 (0.45–0.75)
	GMM	1.34	25 (15%)	0.63 (0.48–0.78)
	High K-means	1.43	22 (14%)	0.60 (0.44–0.75)

Cohen’s kappa was used to determine the degree of concordance between visual assessment and the six different thresholds

*BP*_*ND*_ binding potential, *GMM* Gaussian mixture modelling, *SUVr* standardized uptake value ratio

### Amyloid positivity thresholds in relation to memory slopes

We investigated the association between different definitions of amyloid positivity and memory slopes. We found each operationalization of amyloid positivity was associated with rate of decline on the RAVLT delayed recall (Table 3). Models in which A+ was defined by visual assessment or BP_ND_ thresholds performed somewhat better than models based on SUVr (i.e. lower AIC values).

**Table 3:** Relationship between different amyloid positivity thresholds and longitudinal performance on a memory task

	Threshold	Amyloid status	Estimated annual change	AIC
Visual assessment		Negative	0.19 (0.05)	2933.7
		Positive	− 0.28 (0.09)**	
BP_ND_	0.19	Negative	0.19 (0.05)	2938.5
		Positive	− 0.22 (0.08)**	
	0.23	Negative	0.17 (0.05)	2935.8
		Positive	− 0.28 (0.11)**	
	0.29	Negative	0.15 (0.05)	2938.2
		Positive	− 0.28 (0.12)**	
SUVr	1.28	Negative	0.16 (0.05)	2941.2
		Positive	− 0.21 (0.10)**	
	1.34	Negative	0.14 (0.05)	2940.0
		Positive	− 0.28 (0.12)**	
	1.43	Negative	0.14 (0.05)	2943.0
		Positive	− 0.29 (0.13)**	

Values given are beta (SE), as estimated by linear mixed models (predictor: amyloid status, outcome: score on RAVLT delayed recall). Numbers reflect annual change in raw score points. Models are adjusted for age, sex, education and scanner type

*AIC* Akaike information criterion, *SE* standard error, *RAVLT* Rey auditory verbal learning task, *BP*_*ND*_ binding potential, *SUVr* standardized uptake value ratio

^**^p value < 0.01. *p* value represents the significance of the difference between a positive amyloid status compared with a negative amyloid status

### Relationship between grey zone amyloid burden and memory slope

**Fig. 2:**
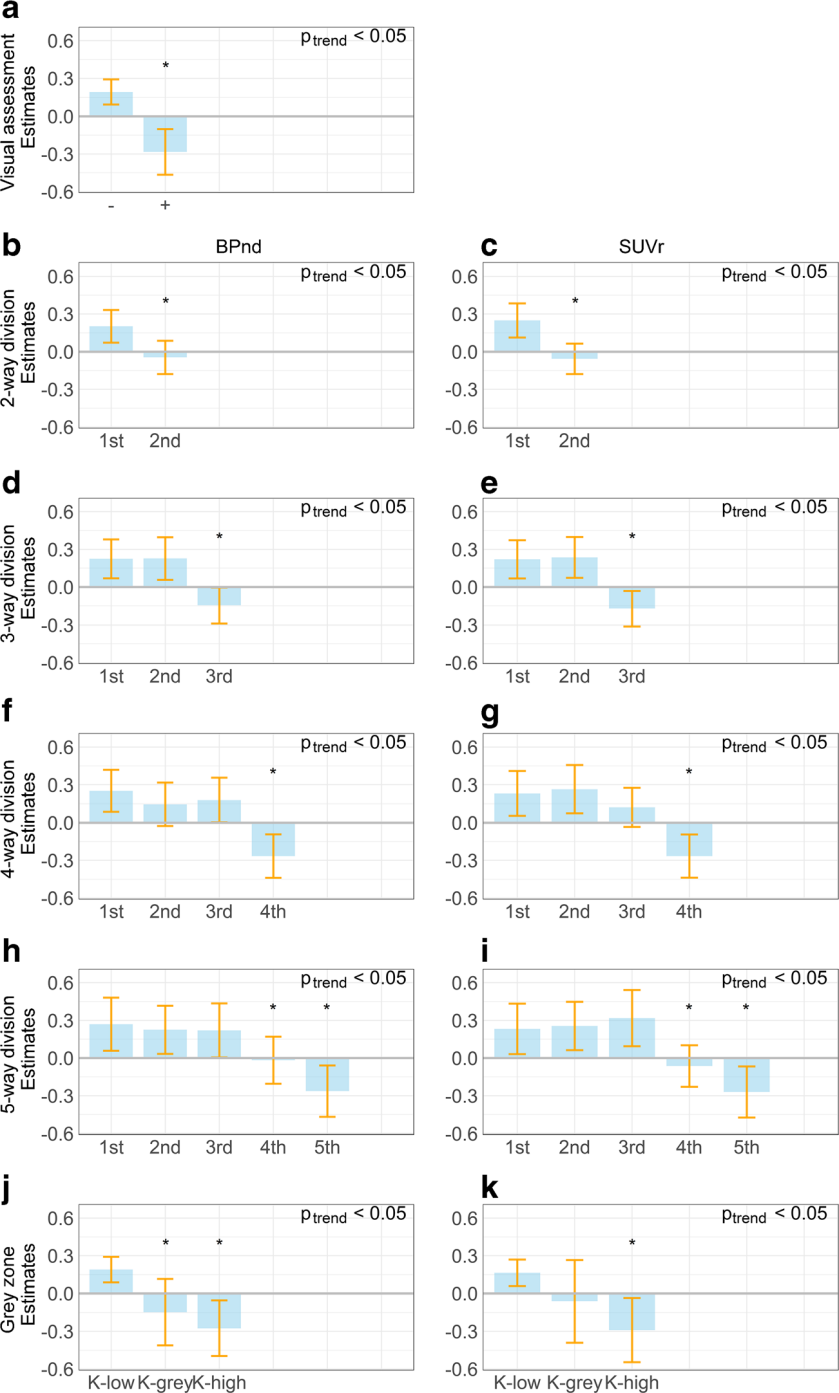
Estimated longitudinal change on RAVLT delayed recall. Bar graphs showing estimated longitudinal change for performance on RAVLT delayed recall over time. The sample was divided into subgroups using visual assessment (**a**), an increasing number of quantiles (**b**–**i**), and the predefined grey zone (**j**–**k**). **a** Visual assessment. **b** Two-way division (BP_ND_). **c** Two-way division (SUVr). **d** Three-way division (BP_ND_). **e** Three-way division (SUVr). **f** Four-way division (BP_ND_). **g** Four-way division (SUVr). **h** Five-way division (BP_ND_). **i** Five-way division (SUVr). **j** Grey zone (according to K-means thresholds) (BP_ND_). **k** Grey zone (SUVr)). Bars represent predicted annual change in raw test score, and error bars represent 95% confidence interval. *P*_trend_ represents *p* value for trend. *: *p* value represents significance (< 0.05) of the difference between the subgroup under investigation and the reference category within the subgroup division (1st subgroup)

Next, we categorised participants based on an increasing number of quantiles to evaluate whether the association between amyloid burden and memory slope is based on a gradual change in amyloid burden. For all models, a gradually lower annual memory performance is seen with increasing amyloid levels (all *p* for trend < 0.05). Subgroups with the lowest amyloid burden (1st halve, 1st third, 1st quarter, 1st fifth and K-low) showed a practice effect, with an increase in memory performance over time, whilst subgroups with the highest amyloid burden (3rd third, 4th quarter, 5th fifth and K-high) showed memory decline over time (Fig. 2). Memory slopes of individuals in the grey zone were intermediate, with betas closer to zero or negative, shown most clearly in the grey zone (0.19–0.29 BP_ND_) and the 4th fifth (0.14–0.22 BP_ND_, 0.21–1.31 SUVr, Fig. 2).

## Discussion

In this sample of cognitively normal individuals with SCD, we observed that grey zone amyloid burden contains relevant clinical information. Furthermore, we obtained thresholds for amyloid positivity based on both SUVr and BP_ND_, which corresponded well with visual assessment of amyloid deposition.

We investigated the association between grey zone amyloid burden and memory function. We found that cognitively healthy individuals with low amyloid levels showed improved memory performance over time, which could be due to a practice effect. By contrast, individuals with substantial amyloid burden showed memory decline over time. Individuals with grey zone amyloid burden had slopes in between, showing neither decline, nor improvement in memory. This implies that these individuals did not benefit from a practice effect, like amyloid negative individuals do. The absence of a practice effect is not an innocent finding, as it has previously been demonstrated as a predictor of future deterioration [34–37]. Although for all subgroups, the estimated annual change was relatively small, the fact that differences could already be measured in this very early stage provides evidence for the concept of a grey zone. Furthermore, some individuals might already experience a subclinical decline in test scores, whilst the test scores themselves are still within normal limits. This illustrates the relevance of longitudinal research to capture within-subject changes over time. Comparison with other studies is complicated because there is not one universal grey zone definition. Studies that focused on peri- or subthreshold amyloid levels have had different approaches, for example studying amyloid negative subthreshold individuals [25, 26, 38], or CSF/PET discordant cases [39]. In a recent article, the grey zone is proposed as a region of uncertainty around the threshold for which more data is needed to actually estimate the risk of cognitive decline or clinical progression [40]. These previous studies found that individuals in the subthreshold range can be on the path to further neurodegeneration (i.e. atrophy, tau pathology, hypometabolism) [38, 41], and are at risk of further amyloid accumulation, cognitive decline and clinical progression [25, 26, 39]. In the present study, we defined the grey zone making use of two thresholds obtained in a data-driven way. In a second approach, we subdivided the data using divisions based on quantiles. Irrespective of the approach, our findings showed that the negative relationship between amyloid and memory performance is not merely driven by the small number of individuals with high amyloid burden, but rather that the variability in amyloid burden, even within normal limits, has potential clinical value.

We used different data-driven methods, such as Gaussian mixture modelling and K-means clustering, to derive cut-off values for amyloid positivity. We found thresholds of 0.19, 0.23 and 0.29 for BP_ND_, and thresholds of 1.28, 1.34 and 1.43 for SUVr. Literature has generated inconsistent findings with respect to amyloid thresholds, ranging from 1.08 to 1.34 for SUVr, with 1.10 being reported most frequently [12, 18–23, 42–45]. The large variability indicates that thresholds may to some extent rely on methodology, image processing pipeline used and study sample. For example, the partial volume correction method [46] and the choice of ROIs [47] affect the degree of amyloid burden. For this reason, we used a commonly used ‘meta ROI’ [21, 24, 25], which is able to clearly distinguish AD patients from cognitively normal controls. However, small differences can be seen across studies [26, 48, 49]. In addition, thresholds are dependent on sample characteristics [50]. We aimed to minimize this effect with our choice of robust data-driven methods. Although our thresholds seemed substantially higher than the aforementioned thresholds, all corresponded equally well to visual assessment. We show that dichotomized BP_ND_ values may even correspond to visual assessment somewhat better, which is consistent with the findings of a previous [^18^F] flutemetamol PET study [13]. All amyloid positivity thresholds predicted future memory decline, which is consistent with another study [51], although models with BP_ND_ thresholds and visual assessment seemingly resulted in a slightly better fit. Because of the underlying gradual association between amyloid burden and memory function, apparently the height of the threshold does not necessarily have a substantial effect on the association between amyloid positivity and memory function.

Strengths of this study include that we used two measures of amyloid quantification, BP_ND_ and SUVr, and that we applied various data-driven approaches. BP_ND_ has been shown to be less sensitive to differences in flow and we found a good concordance with visual assessment. Using BP_ND_ and SUVr as continuous measures enabled us to thoroughly explore the grey zone, which is not possible with a strict binary division like visual assessment. Furthermore, we had a large, well-defined cohort, with a relatively long follow-up. Limitations include the lack of a gold standard such as pathology confirmation. Notwithstanding, we used visual assessment for comparison analyses which has been shown to correlate very well with pathology [12, 52]. Furthermore, we used memory decline as outcome measure, as opposed to clinical progression to MCI or dementia. This might have led to less robust results because memory performance may be reversible, particularly in cases with limited amyloid burden. On the other hand, it might take a relatively long time before a substantial part of this sample shows clinical progression or cognitive impairment. Nevertheless, since we had cognitive data covering on average 3.8 years, our models should give an accurate estimation of memory slope. Using these methods, we were able to capture subtle decline, and found evidence for a diminished practice effect in grey zone amyloid burden. Last, although BP_ND_ and SUVr are widely used continuous measures, it is difficult to compare tracers and sites due to the large variability in methodology previously mentioned. The aim of the Centiloid Project is to provide a method with which this is possible. In this method, all outcome measures are standardized by scaling them to a 0 to 100 scale [53, 54]. For future research, it would be of interest to explore how our results translate to the Centiloid scale [55].

Our demonstration of the potential significance of grey zone amyloid burden may have several clinical consequences. Especially for individuals with amyloid burden within the grey zone, a single threshold might not be very good at distinguishing individuals with a high and a low risk of cognitive decline [4, 40]. When a binary division is warranted, our results imply that only lower thresholds that include the grey zone capture all individuals at risk of memory decline, which corresponds to a previous study that showed existing thresholds for Pittsburgh compound B (PIB) seem too high [24]. When a high threshold is used (e.g. 0.29 BP_ND_) that classifies 16% as amyloid positive, 9% of individuals that are actually also at risk are labelled amyloid negative. This means a total of 25% of individuals is at risk of future deterioration. When considering that the 4th subgroup of the 5-way division also already demonstrates a diminished practice effect, even up to 40% might be at risk (4th and 5th subgroup together). This could have consequences for clinical trials that only include amyloid positive individuals. Excluding grey zone individuals would lower recruitment rates and means loss of valuable information. In addition, these subjects could benefit the most from disease modifying drugs as they are very early in the disease course.

In summary, we showed that various thresholds correspond well to visual assessment in our sample, particularly BP_ND_ thresholds. We furthermore show that not only a high amyloid burden but also grey zone amyloid burden has an effect on longitudinal memory function. We therefore suggest, when the same methodology is used, to use a low BP_ND_ threshold of 0.19 when a binary classification is needed, to also include the grey zone.

## Data Availability

Any data used within the article may be shared upon reasonable request.
